# The Significance of Removing Ruptured Intervertebral Discs for Interbody Fusion in Treating Thoracic or Lumbar Type B and C Spinal Injuries through a One-Stage Posterior Approach

**DOI:** 10.1371/journal.pone.0097275

**Published:** 2014-05-14

**Authors:** Qian-Shi Zhang, Guo-Hua Lü, Xiao-Bin Wang, Jing Li

**Affiliations:** Department of Spine Surgery, The Second Xiangya Hospital of Central South University, Changsha, Hunan, P. R. China; University of Sheffield, United Kingdom

## Abstract

**Objectives:**

To identify the negative effect on treatment results of reserving damaged intervertebral discs when treating type B and type C spinal fracture-dislocations through a one-stage posterior approach.

**Methods:**

This is a retrospective review of 53 consecutive patients who were treated in our spine surgery center from January 2005 to May 2012 due to severe thoracolumbar spinal fracture-dislocation. The patients in Group A (24 patients) underwent long-segment instrumentation laminectomy with pedicle screw-rod fixators for neural decompression. In Group B (29 patients), the patients underwent long-segment instrumentation laminectomy with pedicle screw-rod fixators for neural decompression evacuating of the ruptured disc and inserting of a bone graft into the evacuated disc space for interbody fusion. The mean time between injury and operation was 4.1 days (range 2–15 days). The clinical, radiologic and complication outcomes were analyzed retrospectively.

**Results:**

Periodic follow-ups were carried out until an affirmative union or treatment failure took place. A progressive kyphosis angle larger than 10°, loss of disc height, pseudoarthrosis, recurrence of dislocation or subluxation, or instrument failure before fusion were considered treatment failures. Treatment failures were detected in 13 cases in Group A (failure rate was 54.2%). In Group B, there were 28 cases in which definitive bone fusion was demonstrated on CT scans, and CT scans of the other cases demonstrated undefined pseudoarthrosis without hardware failure. There were statistically significant differences between the two groups (p<0.001 chi-square test). The neurologic recoveries, assessed by the ASIA scoring system, were not satisfactory for the neural deficit patients in either group, indicating there was no significant difference with regard to neurologic recovery between the two groups (p>0.05 Fisher's exact test).

**Conclusion:**

Intervertebral disc damage is a common characteristic in type B and C spinal fracture-dislocation injuries. The damaged intervertebral disc should be removed and substituted with a bone graft because reserving the damaged disc in situ increases the risk of treatment failure.

## Introduction

In most type B and type C fracture-dislocations in the thoracic and lumbar spine, the intervertebral disc between the two separated segments is almost always injured. Its annular fibrosis is usually torn and ruptured leading to a nucleus pulposus leakage out of the disc space [1]. Furthermore, the anterior and posterior longitudinal ligaments are also ruptured in severe dislocation cases [2]. In many relevant literature reports, only a few authors discussed the ruptured intervertebral disc and its care [3]. If the injured disc is ignored and left in the spine, we hypothesized that it may have an adverse effect on the prognosis of the spinal fracture and dislocation. Although a few non-spine professionals have noticed the close relationship between spinal fractures or dislocations and disc injuries [4]–[6], there have been few clinical research reports specifically pertaining to the detrimental influence of ruptured discs on successful long-term bony fusion. It is well known that intervertebral discs do not have a blood supply in adults; their metabolism is extremely slow, and their growth potential for self-repair is very weak. Hence, once one is damaged, it has no opportunity to restore its pre-injury strength and function. Thus, the damaged disc interposes between two adjacent vertebrae (one or both of the vertebrae are also injured) and prevents the two vertebrae from contacting tightly, which is a major hindrance to bony fusion in the fracture-dislocation healing process. In our clinical practice, we found there were some recurrent dislocation and hardware breakage patients who suffered spinal fracture-dislocations and only underwent a posterior reduction, instrumentation, and posterolateral fusion without the removal of the ruptured disc. The failed cases motivated us to determine their root causes. Therefore, we decided to add the procedure of removing the ruptured intervertebral discs during the latter part of our practice and conducted a comparative investigation to determine whether reserving the damaged intervertebral disc is a source of treatment failure.

## Methods and Materials

Participants provided their written informed consent to participate in this study and this study was approved by the Institutional Review Board (IRB) of The Second Xiangya Hospital, Central South University.

### General information and patient grouping

From January 2005 to May 2012, 53 patients (16 female and 37 male) from 19 to 55 years of age (mean 37.9 years of age), who suffered thoracic or lumbar type B or type C spine fracture-dislocation injuries were admitted to our spinal surgery center. The injuries were caused by a vehicle accident on a highway for 19 patients, falls from significant heights for 13 patients, violent blows from heavy objects for 12 patients, and coal mine collapses for 9 patients. The involved spinal segments included the upper thoracic spine in 11 cases, the thoracolumbar spine (T_10_-L_2_) in 37 cases, and the lumbar spine in 5 cases. The neurological injuries were evaluated according to the ASIA scale and the results were judged as follows: Grade A for 31, Grade B for 12, Grade C for 6, and Grade D for 4 patients. According to the AO classification for spine fractures and dislocations, there were 17 cases ascribed to subtype B_1_, 10 cases to subtype B_2_, 3 cases to subtype B_3_, 8 cases to subtype C_1_, 7 cases to subtype C_2_, and 8 cases to subtype C_3_.

We divided the patients into two groups according to whether the ruptured disc was removed and an intervertebral body fusion was performed. The patients in Group A (24 patients) underwent long-segment instrumentation laminectomy with pedicle screw-rod fixators, for neural decompression. In Group B (29 patients), the patients underwent long-segment instrumentation laminectomy with pedicle screw-rod fixators for neural decompression, evacuation of the ruptured disc, and insertion of a bone graft into the evacuated disc space for interbody fusion. The difference between these two surgical techniques lies in whether the ruptured disc is removed and an intervertebral body fusion with a posterolateral approach is performed. Periodic follow-ups were conducted until affirmative union or treatment failure occurred. The local kyphotic angle change, loss of disc height, instrument failure and non-fusion were recorded and analyzed in each case.

### Patient demographics and spinal trauma severity in each group

In Group A, there were 18 males and 6 females of 19 to 52 years of age (mean 37.6 years of age). The injury types included B1 in 9 cases, B2 in 6, B3 in 1 case, C1 in 2 cases, C2 in 4 cases, and C3 in 2 cases. The neurologic status was judged as Grade A in 14 cases, Grade B in 6 cases, Grade C in 2 cases, and Grade D in 2 cases. The involved spinal segments were located in the upper thoracic levels for 5 cases, in T10-L2 for 17 cases, and in L3 for 2 cases. In Group B, there were 19 males and 10 females of 22 to 54 years of age (mean 38.1 years of age). The injury types included B1 in 9 cases, B2 in 4 cases, B3 in 2 cases, C1 in 4 cases, C2 in 4 cases, and C3 in 6 cases. The neurologic status was judged as Grade A in 17 cases, Grade B in 6 cases, Grade C in 4 cases, and Grade D in 2 cases. The involved spinal segments were located in the upper thoracic levels for 6 cases, in T10-L2 for 20 cases, and in L3 for 3 cases. The demographic characteristics of the two groups are shown in table 1.

**Table 1:** Demographic characteristics of the two groups.

	Group A	Group B	Total
Patients	24	29	53
Male	18	19	37
Female	6	10	16
Mean age(range)*#	37.6±11.4(19–52)	38.1±10.7(22–54)	37.9±10.9(19–54)
Level of fracture			
upper thoracic spine	5	6	11
thoracolumbar	17	20	37
lumbar	2	3	5
Cause of injury			
vehicle accidents	8	11	19
falls from heights	7	6	13
violent blows from heavy objects	4	8	12
coal mine collapses	5	4	9
ASIA scale pre-operative			
A	14	17	31
B	6	6	12
C	2	4	6
D	2	2	4
E	0	0	0
AO classification			
B1	9	9	18
B2	6	4	10
B3	1	2	3
C1	2	4	6
C2	4	4	8
C3	2	6	8
Associated injuries			
rib fracture	13	12	25
pulmonary contusion	0	1	1
abrasion of skin	19	27	46
time between injury and operation #	3.9(range 3–15)	4.2(range 2–13)	4.1(range 2–15)

*Units: years.

#P>0.05 t test.

### Surgical treatment

The associated injuries included extremity fractures, rib fractures, pulmonary contusions (one patient), and abrasions of skin. The mean time between injury and operation was 4.1 days (range, 2–15 days). The vertebral segment was stabilized by bilateral pedicle fixation 2 or 3 levels above and 2 levels below the fracture. Five to six segments were fixed using 8 to 10 pedicle screws. There was no significant difference in the method of bilateral pedicle fixation between the two groups(p>0.05) (Table 2). While exposing layer by layer, the local anatomic changes were observed and the existence of locked or jumped articular facets or articular process fractures was identified. After completing routine screw implantation and then releasing the locked facets by resecting the tip of articular processes, the rods were inserted into the screw ends, and the cap nuts were gradually screwed into the screw ends to lock the rods one after another from the most caudal to the most cephalic screw. In the process of tightening the cap nuts, a pulling force was produced spontaneously to lift the upper dislocated spine backward until it reduced completely. If complete reduction was not obtained at first reduction, then the depth of the pedicle screws was adjusted and/or the locked articular processes were further released, and good alignment was finally achieved. Because neurological injuries were present in all of the patients, exploration and decompression were performed routinely by resecting one upper lamina and one lower lamina adjacent to the displaced disc with a Kerrison rongeur, and the local autograft was saved for interbody fusion. If disc or bone fragments were detected in the spinal canal, they could be removed through a posterolateral approach. The patients in group A (N = 24) only underwent posterolateral routine fusion. The patients in group B (N = 29) underwent ruptured disc evacuation and interbody fusion using a posterolateral approach.

**Table 2:** Comparison of fixation segment in the two groups.

	Group A	Group B
Five segments	10	12
Six segments	14	17
Total	24	29

T test was used for statistical analysis showing there was no significant difference between the two groups, p>0.05.

The concrete operative steps to remove the damaged disc and perform interbody fusion by a posterolateral approach were as follows: the decompression scope was extended by removing the relevant articular processes and facets on both sides to open the foramina and expose the lateral parts of the disc; the dura was gently moved from the working side slightly so that the ipsilateral half of the disc space could be visualized clearly; and if veins were covering the disc space, a bipolar coagulator was used to coagulate the vessels to prevent bleeding. Thus, the damaged disc was removed bilaterally through a posterolateral entry without over-traction of the dura tube. The disc tissue, including cartilage endplates and fracture fragments, was removed as thoroughly as possible by using a curette or small sharp osteotome until the cancellous bone was exposed to generate a well-prepared bone graft bed. Then, a local autologous bone graft was packed tightly into the prepared disc space to complete the interbody fusion.

### Postoperative care

Rehabilitation treatment was initiated after 10 to 12 weeks of bed rest after the surgery. Sitting or standing training was initiated first, then the patients started active activities when they were capable under the protection of an external orthosis. The periodic follow-up interval was three months, but the patients could return sooner for further workups if an abnormal condition was felt. 3D CT scans were taken at 12 months postoperatively for the eventless patients to determine whether a solid fusion had been achieved.

## Results

There were no significant differences between the two groups regarding age and time between injury and operation. The average operation time was 124±19 minutes (range, 93–154 minutes) in group A and 195±28 minutes (range, 168–232 minutes) in group B. The average amount of blood loss was 148±66 mL (range, 50–225 mL) in group A and 480±150 mL (range, 250–750 mL) in group B. There was a significant difference between the two groups with regards to the duration of the operation and the amount of blood loss (p<0.001, t test); the results are shown in Table 3.

**Table 3:** Comparison of operation time and blood loss in the two groups.

	Group A(N = 24)	Group B(N = 29)	T test
operation time	124±19(range, 93–154 minutes)	195±28(range 168–232 minutes)	P<0.001
blood loss	148±66(range 50–225 mL)	480±150(range 250–750 mL)	P<0.001

T test was used for statistical analysis showing that there was a significant difference between the two groups according to the duration of operation and amount of blood loss, p<0.001.

The surgery was considered a failure if the implant was broken before union was achieved or if complications occurred in the patients, including pseudarthrosis and loss of disc height if the radiographs taken at follow-up demonstrated an increase of ≥10° in the sagittal kyphosis compared to the local kyphotic angle measured immediately postoperatively [7], or if dislocation or subluxation was recurrent. Sagittal local kyphosis was measured from the superior endplate of the cephalic intact vertebra to the inferior endplate of the caudal intact vertebra.

In group A, there were 13 cases of sustained failure within one year, consisting of hardware breakage, pseudarthrosis formation, local kyphotic angle increase, or disc height loss. Spontaneous interbody bridging around the injured disc was achieved in 11 cases. The failure rate was as high as 54.2%; in group A, there were 8(33.3%) cases of loss of disc height, 13(54.2%) cases of implant failure, 1(4.2) case of pseudarthrosis, 6(25%) cases of local kyphotic angle change ≥10° at follow-up compared to immediately after the operation, and 6(25%) cases of dislocation. Back pain was found in all of these failure cases and tuberositas of a fixation under the skin could be observed in 4 cases. In group B, there were 28 cases in which definitive bone fusion was demonstrated by CT scans and the fusion rate was 96.6%; the 1 other case (3.4% of group B) demonstrated doubtful pseudoarthrosis without hardware failure or local pain. There were no complications or mortality and back pain was relieved in all of the patients in group B. There are significant differences statistically between the two groups (p<0.001 chi-square test). The changes in kyphotic angle and disc space height in the failed patients in group A were measured and compared. The results shown in Table 4 demonstrate a significant difference between the two time-points of “immediately postoperative” and “initial detection of failure” for those two parameters.

**Table 4:** Complications in the failed patients in group A.

NO.	Hard ware breakage	Local kyphosis angle (°)			non-union	pseudarthrosis	Disc height (cm)		dislocation	pain
		Immediately postoperative	Initial detection of failure	>10°			Immediately postoperative	Initial detection of failure		
1	+	−12.9	−7.6	-	+	-	0.49	0.33	-	+
2	+	−8.0	−5.8	-	+	-	0.53	0.37	-	+
3	+	−12.8	3.8	+	+	-	0.43	0.28	+	+
4	+	−14.5	−7.5	-	+	-	0.48	0.43	-	+
5	+	−10.0	8.3	+	+	-	0.71	0.34	+	+
6	+	2.5	9.7	-	+	+	0.46	0.18	-	+
7	+	−3.3	12.6	+	+	-	0.38	0.32	+	+
8	+	−3.8	5.6	-	+	-	0.29	0.20	-	+
9	+	2.5	17.3	+	+	-	0.47	0.34	+	+
10	+	−17.2	5.8	+	+	-	0.47	0.39	+	+
11	+	−2.3	5.6	-	+	-	0.30	0.22	-	+
12	+	−11.6	2.0	+	+	-	0.48	0.25	+	+
13	+	−7.6	−5.4	-	+	-	0.41	0.17	-	+

Mann-Whitney test was used for statistical analysis showing significant difference in kyphotic angle and disc height changes between two time-points of “immediately postoperative” and “initial detection of failure”, P<0.001.

Neurologic recoveries were not satisfactory for complete neural deficit patients in either group; in other words, 31 cases that were scored as Grade A prior to the surgery in both groups gained no measurable improvement at the final follow-up. 4 out of 6 Grade B cases in group A improved to Grade C, while the other 2 cases remained Grade B. 4 out of 6 Grade B cases in group B improved to Grade C, and the other 2 cases remained Grade B. 1 out of 2 Grade C cases in group A recovered to Grade D, and 2 cases of Grade C in group B improved to Grade D. 2 Grade D cases in group A recovered to Grade E, and 2 cases of Grade D in group B improved to Grade E. There was no significant difference between the two groups with regard to neurologic recovery (p>0.05 Fisher's exact test). The results are shown in Table 5.

**Table 5:** Neurologic recoveries in the two groups.

	Group A			Group B		
ASIA scale	Immediately postoperative	Initial detection of failure	Cases of improvement	Immediately postoperative	Initial detection of failure	Cases of improvement
A	14	14	0	17	17	0
B	6	2	4	6	2	4
C	2	1+4	1	4	2+4	2
D	2	0+1	2	2	0+2	2
E	0	2	0	0	2	0

Fisher's exact test was used for statistical analysis showing there was no significant difference between the two groups, p>0.05.

## Discussion

Except for the type B2.1 injury (according to the AO classification system), all other type B and C fracture-dislocations in the thoracic and lumbar spine are involved in intervertebral disc rupture and the dominant common feature in these types of injuries is the dislocation along the ruptured disc; that is to say the disc injury is an important component of spinal fracture-dislocations. Compared with type A spinal fractures, type B and C fracture-dislocations signify more severe trauma because of the accompanying complete disruption of the disco-ligamentous complexes, which contribute significantly to maintaining the dynamic stability of the spinal column. Greater violence or higher energy is needed to produce such crippling or devastating spinal column fracture-dislocations and results in a higher degree of neurological injury. Complete paralysis or spinal cord disruption, which is the most severe type of neural injury, is more common in type B and C than in type A injuries. The treatment goals and strategies are also different between these types of injuries and are worth further investigation and clarification because these issues are still controversial.

The treatment goals for type B and C spinal injuries include the following: 1. Re-aligning the dislocated spine to a normal arrangement by simple and effective methods and maintaining this restored alignment for an adequate duration until bony fusion. 2. Sufficient decompression of the spinal cord or cauda equina. 3. Reliable bone grafting to ensure bony fusion which is the ultimate goal — permanent stability should be achieved. A discussion about commonly used treatment strategies is developed below regarding the three goals.

**Figure 1:**
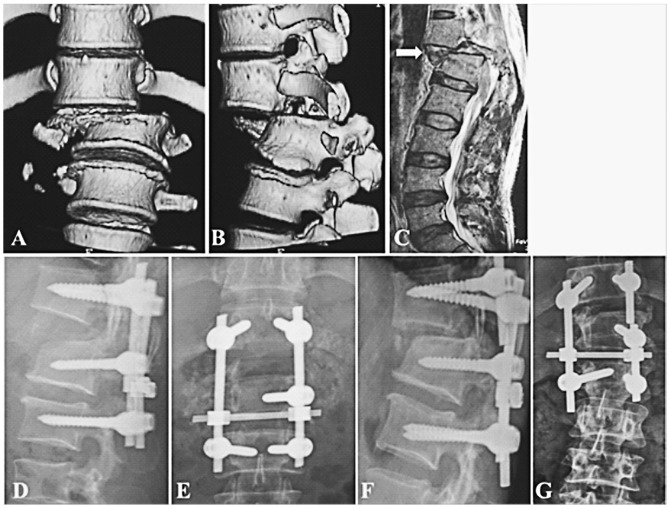
Type C2.2.2 fracture-dislocation in T12-L1 (A, B), the disruption crossing T12-L1 disc and the shattered disc(C, white arrow). Initial operative treatment was given by another hospital with posterior short-segment instrumentation, but the reduction was not complete (D). 9 months later, one rod was broken and disc space was collapsed; kyphosis and scoliosis occurred (F, C).

**Figure 2:**
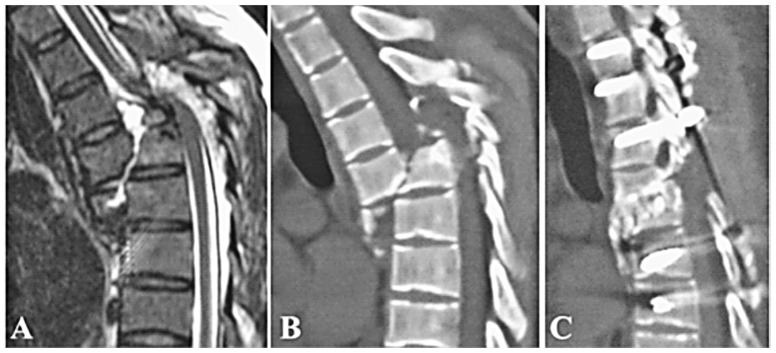
Type B1.2.3 severe fracture-dislocation involving T5-6; the disc was squeezed out of disc space posteriorly (black arrow) and anteriorly (white arrow) and intervened between two fractured vertebrae (A,B). Posterior reduction, instrumentation with 9 pedicle-screws, decompression, T5-6 disc excision, and bone graft in disc space were performed at one stage. One year later, follow-up CT showed good alignment and bony fusion (C).

**Figure 3:**
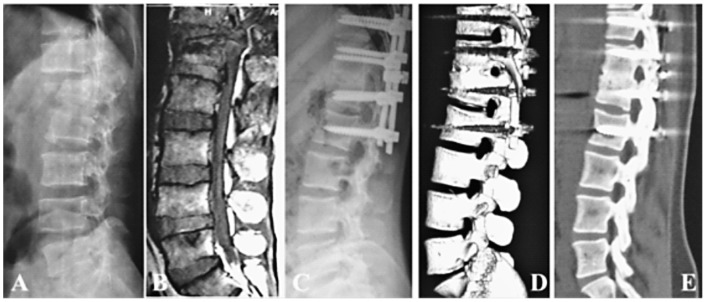
Type B1.2.2 fracture-dislocation in T12-L1 segment with disc rupture (A, B), posterior long-segment instrumentation, decompression, disc excision, and autologous local morselized bone graft in disc space in single-stage (C). Postoperatively 2 years, CT follow-up showing solid fusion in T12-L1 disc space (D, E).

Spinal dislocation and concomitant local kyphotic deformity are the dominant abnormal anatomic changes in type B and C injuries. Posterior surgery is indispensable in that only posterior instrumentation can provide sufficient pulling and lifting strength to reduce dislocation and correct the kyphotic deformity; this major advantage of the posterior approach has been verified by many authors [1], [8], [9]. The pedicle screw is the strongest and most powerful internal fixator available, it has been used since 1959 and is widely accepted. In the process of reducing the anteriorly or laterally dislocated upper part of the spine, a pulling force is needed to lift back the spine segment cephalic to the dislocation site for anatomical reduction by the upper screws and correspondingly, an enormous pullout stress is generated and concentrated on the lower screws. If only four screws are implanted when treating type B and C fracture-dislocations as is done with short-segment fixation in the posterior operation for type A fractures, each screw will sustain greater stress leading to a higher possibility of screw or rod breakage. In addition, four-screw short-segment fixation usually incurs incomplete reduction due to its weaker capability for reduction (Fig. 1). Yu et al reported a failure rate as high as 60% using a short-segment fixation technique [10]. Long-segment instrumentation is now a popular modality for treating such traumas because of its obvious advantages over short-segment fixation in mechanical strength [9]. The case illustrated in Figure 1 was treated by another hospital with 5 pedicle screws and an incomplete reduction was left, which suggests that the short-segment instrumentation was not a dependable method for the reduction of the spinal dislocation in that patient. In the current case series, 8 to 10 pedicle screws were implanted and a complete reduction was achieved in all cases (Fig. 2,3).

Sufficient decompression of the spinal cord or cauda equina can be obtained in several ways. The first method is complete reduction to restore the continuity of the spinal canal, a fundamental step that can regain effective space and relieve tension on the neural structures. The second method is removing the posterior fracture fragments that collapsed into the spinal canal from the vertebral arch by resecting the lamina or articular processes. The third method is removing the anterior compression through a posterolateral approach. This is a demanding technique because anterior compression mainly originates from retropulsed fracture fragments or a ruptured disc located in the front of the neural elements, but it is still a good way to eliminate the anterior compression without additional injury and has been mastered by an increasing number of surgeons.

**Figure 4:**
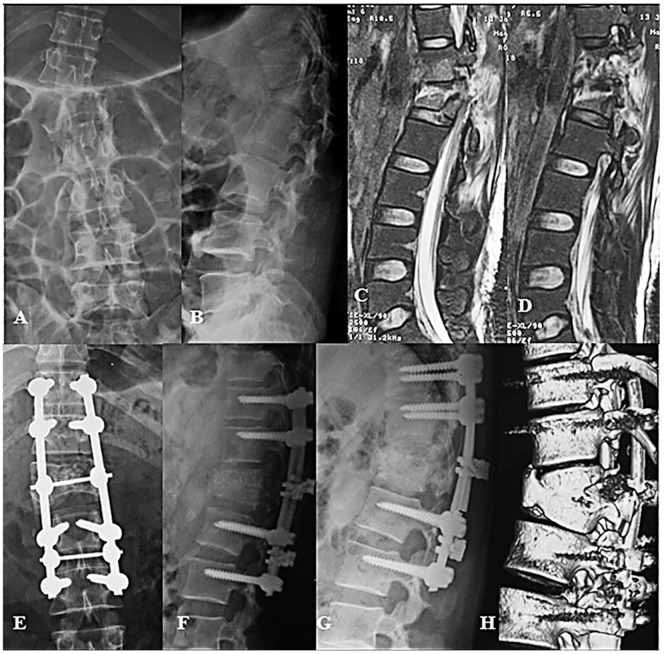
Type C1.3.1 fracture in L1 complicated with its two end-plates and T12-L1, L1-L2 disc rupture (A, B, C, D); excellent posterior reduction and instrumentation were achieved through the operation, but the ruptured discs were left untouched (E, F). Follow-up of 6 months after operation showed one rod was broken, two adjacent discs collapsed, and local kyphotic angle increased(G, H).

To stabilize the discontinuous spine into an integral column, the only permanent method is to achieve a solid bony fusion or reliable healing of the ruptured ligaments and discs to bridge the separated upper and lower segments. Spinal ligaments and discs are well known to have a minimal blood supply and without a blood supply, no human tissue can repair itself. Thus, the self-repair capabilities of disrupted discs and ligaments are weak and unreliable, so the restoration of spinal stability after dislocation cannot be dependent on the self-healing of soft tissues. Even if healing occurs, scar tissue connection is dominant and is not strong enough to maintain spinal stability [5]. At worst, the residual damaged disc within the intervertebral space continues to deteriorate because of accelerated desiccation and degeneration. This results in a greater disc space and height loss [11], [12] which undermines the supportive function of the anterior spinal column and transfers the overloading to the internal fixators. Therefore, disc injury in spinal fracture-dislocation is not only an acute incident but also a chronic sequence of changes over time. Several authors have reported that in some conservatively treated cases, the injured discs collapsed and narrowed gradually, resulting in a late-onset kyphotic deformity because of rapid desiccation and biochemical changes in the nucleus pulposus secondary to the annulus fibrosus damage[5], [13], [14]. Thus, if a ruptured disc is left in situ rather than being removed, it will still interpose between the two separated segments and deter the natural post-traumatic healing process. An unstable spine is put at a disadvantage for fusion, so treatment failure is inevitable in these cases (Fig. 1, 4). Thus, leaving a severely damaged disc in situ is not reasonable; this is the main culprit for treatment failure in these spinal surgeries and it is wise to evacuate the damaged residual disc completely to eliminate its long-term negative effects. Another large benefit of radical disc excision is that it provides an adequate bone graft bed, which is a crucial prerequisite for reliable solid fusion. To best take advantage of this graft bed, adequate preparation should be undertaken in advance, including radically removing the upper and lower cartilage endplates to expose the subcortical cancellous bone in a large enough area (more than 75% of the disc area, or 2×2 cm^2^). Thorough removal of the ruptured disc is a demanding technique because of the limited working field. Partially removing the disc tissue by laminectomy or laminotomy with a posterior approach is a routine operation of the lower lumbar spine; however, the complete removal of the disc by these techniques is difficult, especially for the removal of cartilage endplates, because the surgical field is limited and the surgical instruments, such as curettes and fine osteotomes, cannot be introduced into the disc space via the spinal canal. Thus, extended exposure is necessary by total laminectomy or even facet resection to enlarge the lateral exposure, allowing complete disc excision and avoiding violating the neural elements under direct vision. This approach is also more convenient for removing bone or retropulsing disc fragments to the spinal canal from the vertebral body or disc. We do not think diskectomy and associated facetectomy will increase the risk of instability of the spine in the immediate post-operative period. First, only a ruptured intervertebral disc, which is an unstable factor intrinsically, requires diskectomy. Second, most thoracic or lumbar type B and C fractures have an intrinsically fractured posterior column. In some cases, we can even easily remove the processus articularis without a facetectomy. Lastly, rigid internal fixation can provide enough holding power before a rigid fusion has formed. There was an operative procedure introduced by Daniaux in 1986 of transpedicular intervertebral bone grafting after posterior stabilization of thoracolumbar fractures [15]; the intention of this technique was to remove the ruptured intervertebral disc and refill the space with a bone graft to obtain solid fusion and to prevent the late collapse of the intervertebral disc space, which would incur a recurrent kyphotic deformity and treatment failure. The notion itself was correct, but the clinical outcomes reported by many researchers were not improved compared with a simple posterior stabilization treatment [16], [17]. Why were the results so frustrating? Why did this theoretically reasonable treatment not have better results? The main issue was that the disc could not be resected radically through the very limited transpedicular approach, so the cartilage endplates were not removed and as a consequence, the bone graft bed had a poor blood supply. Although a sufficiently sized bone graft was packed into the disc space, new bone growth could not be induced because of the isolation of the graft from the blood circulation. Naturally, this procedure was doomed to failure and was phased out. To overcome the issue of a poor blood supply to the bone graft bed, we undertook reliable measures for expanding the exposure to ensure an easy access to the injured disc from multiple directions and angles. This approach guaranteed that most of the disc was excised and a good bone graft bed with sufficient blood supply was prepared. By using this surgical procedure in our cases of group B cases, solid bony fusion between two vertebral bodies was achieved in 28 out of 29 patients (Fig. 2,3). However in group A, the ruptured disc was left in situ, and the prognosis of these patients was unsatisfactory with screw or rod breakage or recurrent kyphosis in 13 patients within 1 year postoperatively. There is some doubt regarding the osteogenic capacity and supporting function of the locally obtained autologous morselized bone because its growth into a solid mass from numerous separate, small-sized bone fragments can be a chronic and complicated process. In fact, we initially were not sure whether bony fusion would occur, but we believed it was worth trying. With the accumulation of cases and experience, we finally confirmed that the fusion was sure and solid with this procedure (Fig. 3). In some recent literature papers regarding the treatment of thoracolumbar fracture-dislocation, the authors still only emphasized reduction and instrumentation and did not mention the ruptured intervertebral disc and interbody fusion [18], [19].

In severe thoracolumbar spinal fracture-dislocation cases, we preferred anterior decompression, reconstruction, and stabilization from a one-stage posterolateral approach to a combined anterior-posterior approach. Some authors have preferred a combined anterior-posterior approach because they believe that anterior reconstruction and bone grafting provided by a combined approach are more reliable [20]. In recent literature, authors compared the medium and long-term outcomes of anteroposterior versus posterior approach with corpectomy, decompression, and reconstruction of spine in the treatment of thoracolumbar fractures and showed that the two approaches were both adequate surgical treatments for thoracolumbar fractures. However, the one-stage posterolateral approach has the major advantages of less perioperative volume of blood loss, shorter operative duration, and better pulmonary function[21], [22]. In addition, a combined anterior-posterior approach is more traumatic because of the additional incision, longer hospital stay, greater expense, and complications due to the anterior approach. Particularly in those patients who suffer from multiple traumas or internal diseases, a combined operation can increase mortality [23]. Although the one-stage posterolateral approach will make more damages to the processus articularis, it will not increase the risk of instability of the spine in the immediate post-operative period as mentioned above.

In fact, the surgical technique used for group B was neither advanced nor demanding. Our purpose in conducting this investigation was mainly to determine the negative influence of a damaged disc on long-term spinal stability if it is retained in situ. The aim of this paper is to increase awareness of the facts that the damaged intervertebral disc should not be ignored and that it is necessary to remove the damaged disc as completely as possible to complete a reliable interbody fusion.

## Conclusions

The following conclusion can be drawn from this study: the residual ruptured intervertebral discs should be removed and replaced with a bone graft because reserving the damaged disc in situ is a high risk factor for treatment failure in thoracic and lumbar spinal type B and C fracture-dislocations. Although removing ruptured intervertebral discs leads to a longer operation time and more blood loss, it is still a reliable approach for operation.

## References

[1] Magerl F, Aebi M, Gertzbein SD, Harms J, Nazarian S (1994) A comprehensive classification of thoracic and lumbar injuries. Eur Spine J 3: 184–201. 7866834 10.1007/BF02221591

[2] Elgafy H, Bellabarba C (2007) Three-column ligamentous extension injury of the thoracic spine: a case report and review of the literature. Spine (Phila Pa 1976) 32: E785–788. 18245995 10.1097/BRS.0b013e31815b60fd

[3] Moore TA, Steinmetz MP, Anderson PA (2011) Novel reduction technique for thoracolumbar fracture-dislocations. J Neurosurg Spine 15: 675–7. 21923237 10.3171/2011.8.SPINE1129

[4] Willen JA, Gaekwad UH, Kakulas BA (1989) Burst fractures in the thoracic and lumbar spine. A clinico-neuropathologic analysis. Spine (Phila Pa 1976) 14: 1316–1323. 2617361 10.1097/00007632-198912000-00008

[5] Oner FC, vd Rijt RH, Ramos LM, Groen GJ, Verbout AJ, et al (1999) Correlation of MR images of disc injuries with anatomic sections in experimental thoracolumbar spine fractures. Eur Spine J 8: 194–198. 10413344 10.1007/s005860050156 PMC3611165

[6] Oner FC, der Rijt RR v, Ramos LM, Dhert WJ, Verbout AJ (1998) Changes in the disc space after fractures of the thoracolumbar spine. J Bone Joint Surg Br 80: 833–839. 9768894 10.1302/0301-620x.80b5.8830

[7] Alanay A, Acaroglu E, Yazici M, Oznur A, Surat A (2001) Short-segment pedicle instrumentation of thoracolumbar burst fractures: does transpedicular intracorporeal grafting prevent early failure? Spine 26: 213–7. 11154543 10.1097/00007632-200101150-00017

[8] Mikles MR, Stchur RP, Graziano GP (2004) Posterior instrumentation for thoracolumbar fractures. J Am Acad Orthop Surg 12: 424–435. 15615508 10.5435/00124635-200411000-00007

[9] Inamasu J, Guiot BH, Nakatsukasa M (2008) Posterior instrumentation surgery for thoracolumbar junction injury causing neurologic deficit. Neurol Med Chir (Tokyo) 48: 15–21. 18219187 10.2176/nmc.48.15

[10] Yu SW, Fang KF, Tseng IC, Chiu YL, Chen WJ, et al (2002) Surgical outcomes of short-segment fixation for thoracolumbar fracture dislocation. Chang Gung Med J 25: 253–259. 12079159

[11] Xu BS, Tang TS, Yang HL (2009) Long-term results of thoracolumbar and lumbar burst fractures after short-segmentpedicle instrumentation, with special reference to implant failure and correctionloss. Orthop Surg 1: 85–93. 22009823 10.1111/j.1757-7861.2009.00022.x PMC6583120

[12] Speth MJ, Oner FC, Kadic MA, de Klerk LW, Verbout AJ (1995) Recurrent kyphosis after posterior stabilization of thoracolumbar fractures. 24 cases treated with a Dick internal fixator followed for 1.5−4 years. Acta Orthop Scand 66: 406–410. 7484118 10.3109/17453679508995575

[13] Przybyla A, Pollintine P, Bedzinski R, Adams MA (2006) Outer annulus tears have less effect than endplate fracture on stress distributions inside intervertebral discs: relevance to disc degeneration. Clin Biomech (Bristol, Avon) 21: 1013–1019. 10.1016/j.clinbiomech.2006.07.003 16956702

[14] Mumford J, Weinstein JN, Spratt KF, Goel VK (1993) Thoracolumbar burst fractures. The clinical efficacy and outcome of nonoperative management. Spine (Phila Pa 1976) 18: 955–970. 8367783

[15] Daniaux H, Seykora P, Genelin A, Lang T, Kathrein A (1991) Application of posterior plating and modifications in thoracolumbar spine injuries. Indication, techniques, and results. Spine (Phila Pa 1976) 16: S125–133. 2028327 10.1097/00007632-199103001-00018

[16] Knop C, Fabian HF, Bastian L, Blauth M (2001) Late results of thoracolumbar fractures after posterior instrumentation and transpedicular bone grafting. Spine (Phila Pa 1976) 26: 88–99. 11148651 10.1097/00007632-200101010-00016

[17] Knop C, Fabian HF, Bastian L, Rosenthal H (2002) Fate of the transpedicular intervertebral bone graft after posterior stabilisation of thoracolumbar fractures. Eur Spine J 11: 251–257. 12107794 10.1007/s00586-001-0360-z PMC3610514

[18] Moore TA, Steinmetz MP, Anderson PA (2011) Novel reduction technique for thoracolumbar fracture-dislocations—Technical note. J Neurosurg Spine 15: 675–677. 21923237 10.3171/2011.8.SPINE1129

[19] Inamasu J, Guiot BH, Nakatsukasa M (2008) Posterior instrumentation surgery for thoracolumbar junction injury causing neurologic deficit. Neurol Med Chir (Tokyo) 48: 18–21. 10.2176/nmc.48.15 18219187

[20] Xia Q, Xu BS, Zhang JD, Miao J (2009) Simultaneous combined anterior and posterior surgery for severe thoracolumbar fracture dislocations. Orthop Surg 1: 28–33. 22009778 10.1111/j.1757-7861.2008.00006.x PMC6734638

[21] Ma Y, Deng SC, Jia ZH, Hao YH (2013) Lateral position one-stage combined anteroposterior approach versus posterior approach with subtotal corpectomy, decompression, and reconstruction of spine in the treatment of thoracolumbar burst fractures. Zhonghua Yi Xue Za Zhi 93: 2112–6. 24284239

[22] Ayberk G, Ozveren MF, Altundal N, Tosun H, Kaplan M, et al (2008) Three column stabilization through posterior approach alone: transpedicular placement of distractable cage with transpedicular screw fixation. Neurol Med Chir (Tokyo) 48: 8–14. 18219186 10.2176/nmc.48.8

[23] P Oprel P, Tuinebreijer WE, Patka P, den Hartog D (2010) Combined anterior-posterior surgery versus posterior surgery for thoracolumbar burst fractures: a systematic review of the literature. Open Orthop J 4: 93–100. 21283533 10.2174/1874325001004010093 PMC3031139

